# Structural Analysis of microRNAs in Myeloid Cancer Reveals Consensus Motifs

**DOI:** 10.3390/genes13071152

**Published:** 2022-06-26

**Authors:** Senol Dogan, Emrulla Spahiu, Anis Cilic

**Affiliations:** 1 Faculty of Physics and Earth Sciences, Peter Debye Institute, Leipzig University, 04103 Leipzig, Germany; 2 Institute of Molecular and Cell Physiology, Hannover Medical School, Carl-Neuberg-Straße 1, 30625 Hannover, Germany; spahiu.emrulla@mh-hannover.de; 3 Excellence Cluster Cardiopulmonary System, University of Giessen and Marburg Lung Center (UGMLC), Justus-Liebig-University, 35392 Giessen, Germany; anis.cilic@innere.med.uni-giessen.de

**Keywords:** microRNA, structure, consensus motifs, miRNA motifs, cancer, gene regulation

## Abstract

MicroRNAs (miRNAs) are short non-coding RNAs that function in post-transcriptional gene silencing and mRNA regulation. Although the number of nucleotides of miRNAs ranges from 17 to 27, they are mostly made up of 22 nucleotides. The expression of miRNAs changes significantly in cancer, causing protein alterations in cancer cells by preventing some genes from being translated into proteins. In this research, a structural analysis of 587 miRNAs that are differentially expressed in myeloid cancer was carried out. Length distribution studies revealed a mean and median of 22 nucleotides, with an average of 21.69 and a variance of 1.65. We performed nucleotide analysis for each position where Uracil was the most observed nucleotide and Adenine the least observed one with 27.8% and 22.6%, respectively. There was a higher frequency of Adenine at the beginning of the sequences when compared to Uracil, which was more frequent at the end of miRNA sequences. The purine content of each implicated miRNA was also assessed. A novel motif analysis script was written to detect the most frequent 3–7 nucleotide (3–7n) long motifs in the miRNA dataset. We detected CUG (42%) as the most frequent 3n motif, CUGC (15%) as a 4n motif, AGUGC (6%) as a 5n motif, AAGUGC (4%) as a 6n motif, and UUUAGAG (4%) as a 7n motif. Thus, in the second part of our study, we further characterized the motifs by analyzing whether these motifs align at certain consensus sequences in our miRNA dataset, whether certain motifs target the same genes, and whether these motifs are conserved within other species. This thorough structural study of miRNA sequences provides a novel strategy to study the implications of miRNAs in health and disease. A better understanding of miRNA structure is crucial to developing therapeutic settings.

## 1. Introduction

MicroRNAs (miRNAs) are single-stranded non-coding RNAs made up of short nucleotide sequences with lengths varying between 17 and 27 nucleotides, the vast majority being 20–21 nucleotides long [1,2]. Although miRNAs are relatively short sequences, they are effective enough to function as gene handbrakes and prevent long transcripts from being translated into proteins. MiRNAs interact with specific parts of a transcript by base-pairing [3]. 

MiRNAs play a crucial role in the maintenance of the homeostasis of important metabolic pathways and processes [4]. The alteration of miRNA levels is associated with cancer and developmental biology [5]. An up- or downregulation of miRNA expression serves as an effective reason for the development and spread of cancer [6]. The changing amount of protein in a cell by miRNA regulation affects the molecular function and harmony of the cell [7]. While the amount of intracellular protein is directly related to the expression of genes, it can also be indirectly affected by miRNA expression by inactivating the target genes before being translated into proteins [8]. This problem leads to less effective protein synthesis than the required amount in cancer cells and this in turn drives them to act according to cancers’ constitution [9]. In a cancer cell, while the gene expression is directly altered via mutations or methylations, it is indirectly inactivated with the expression of miRNAs [10]. Research in fields other than cancer, such as cell developmental biology, stem cell, and cardiovascular research has also shown that the cell’s miRNA expression affects the deactivation of some biological mechanisms [11,12]. The impact of such miRNA-based control on gene expression makes miRNAs one of the important epigenetic factors that could be used effectively as therapeutic targets in translational research [13,14].

Although miRNA genes are very short compared to genes coding for proteins, they are transcribed like genes and fulfill their functions using complementary base pairing. They function as a part of the ribonucleoprotein complex RISC (RNA-induced silencing complex) and by binding to the complementary target, they potentiate the action of the RISC [15]. The nucleotides at positions 2 to 8 near the 5′ end of miRNAs are predominantly binding sites of miRNAs and are called seed regions [16]. In some forms of binding, seed complementarity is not enough in itself and requires pairing in the central or end region of the miRNAs [17]. In most cases, miRNAs interact with the 3′ untranslated regions (3′ UTR) of target mRNAs to induce mRNA degradation and translational repression [2]. This striking positional affinity has led to the development of miRNA target search algorithms that focus on 3′UTRs for further amplification of the bias for functional 3′UTR sites [18]. However, effective miRNA-binding sites have also been identified in the 5′UTR or the open reading frame (ORF) of target mRNAs [19,20,21,22]. Different studies and computer tools that measure and reveal the miRNA-gene relationship are expressed in different ways [23]. In this work, our first question was to find the miRNAs with similar nucleotide sequences and the second one was if the extent of the similarity would give us information about the consensus miRNAs and their target proteins [24].

## 2. Materials and Methods

**Figure 1:**
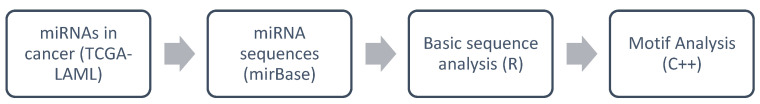
Flowchart of data collection and processing. In brackets is the database/tool used to perform the analysis.

The main purpose of the research was to analyze the nucleotide sequences and specific motifs of microRNAs implicated in myeloid cancer. To achieve this, the miRNA-seq data were collected and structured from different databases for analysis, as described in the workflow chart in Figure 1.

We used GDC Data Portal to find the miRNAs that are most frequently altered in myeloid cancer (https://portal.gdc.cancer.gov/) (accessed on 25 April 2016) [25]. Specifically, the TCGA project for Acute Myeloid Leukemia (TCGA-LAML) was used under the filters transcriptome profiling, miRNA Expression Quantification, miRNA-Seq, and BCGSC miRNA Profiling. The dataset was downloaded in May 2021 and consists of the microRNA expression levels of the 197 AML patients determined by Illumina HiSeq 2000 microRNA seq. The level 3 sequencing data (expression levels of each miRNA) into the Log2 scale were used [26]. The set of isoform.quantification.txt files, which give read counts at base-pair resolution, contained the total read counts for mature miRNA (corresponding to miRBase v13 MIMAT identifiers), normalized to RPM. 

Next, the nucleotide sequences of these miRNAs were retrieved manually from the miRBase database (https://www.mirbase.org/) (accessed on 1 February 2022) [27] to use in sequence and motif analysis. The nucleotide length of these miRNAs was plotted in a histogram, followed by the conduct of descriptive statistics using R Studio [28]. Next, the nucleotide frequency of each position was assessed together with purine/pyrimidine content using an Excel spreadsheet. In the second part of the project, we wrote a C^++^ script to analyze the miRNA sequences and identify the motifs in miRNAs in cancer (code deposited in GitHub [29]). 

To identify the target genes of the miRNA that contain the consensus motifs, we first downloaded the validated target genes database from the mirTarBase database [30]. Target genes of all the sequences containing the motif of interest were taken from this database (for each motif separately). A total number of target genes (how many genes are targeted by the sequences with the motif) and the gene frequency (the number of sequences with the motif targeting the same gene) were calculated (for each motif separately).

Finally, to search for the conservation of our motifs, we downloaded the mature mRNA sequences from different species using mirbase.org. All of the sequences for selected species were used and analyzed for motifs with our program written in C^++^. Databases with 5n and 6n motif frequencies for each species were developed and the motif frequency was derived showing how many miRNA sequences (from one species) contain the motifs.

## 3. Results

The data retrieved from GDC Portal were from the miRNA profiling of patients with hematopoietic and reticuloendothelial cancer. This yielded a dataset of 587 miRNAs elevated in myeloid cancers. The sequences of these miRNAs were extracted from miRBase, a sample of data is given in Table 1.

### 3.1. miRNA Sequence Length and Nucleotide Frequency Analysis

**Figure 2:**
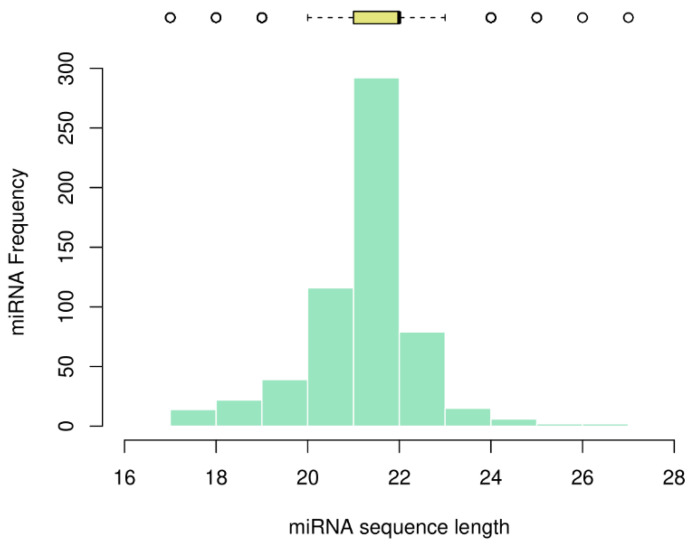
The distribution of miRNA length in myeloid cancer.

We analyzed the sequence length of the miRNAs and plotted the frequency as a histogram, together with the descriptive statistics as a boxplot on top (Figure 2).

Most miRNAs in myeloid cancer were found to be 21, 22, and 23 nucleotides in length, with percentages of 19.8%, 49.8%, and 13.5%, respectively. We calculated a mode and median of 22 nucleotides, with an average of 21.69 and a variance of 1.65. The shortest miRNAs consist of 17 nucleotides (namely, hsa-mir-1260, hsa-mir-1825, hsa-mir-1207, hsa-mir-453, hsa-mir-1268, hsa-mir-1306, hsa-mir-1321, and hsa-mir-1827) and the four longest miRNAs consist of 26 and 27 nucleotides (hsa-mir-1248, hsa-mir-1183, hsa-mir-1272, and hsa-mir-1244). 

It is not yet known whether the nucleotide length of miRNAs plays an active role in cancer or other diseases. Perfect base-pairing leads to the degradation of mRNA (a mechanism mainly seen in plants) and imperfect base-pairing with the target mRNA leads to repression of translation [31]. In this line of argumentation, it could be assumed that the longer the miRNA sequence, the higher the probability to complement the target mRNAs. However, this needs experimental proof. Based on the volume of literature published for each miRNA in miRBase, we noted a higher volume of research carried out on short miRNAs (consisting of 17 and 18 nucleotides) when compared to the ones with 26 and 27 nucleotides (Table 2).

**Figure 3:**
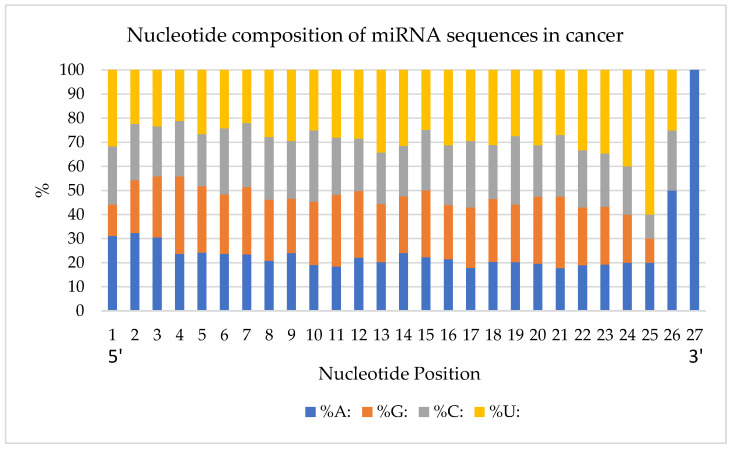
Percentage of nucleotides in each position of miRNAs studied. Position 1–27 corresponds to direction 5′-3′.

Next, the percentage of nucleotides in each position was quantified (Figure 3). The analysis was done from the first (5′) to the last (3′) position of the miRNA’s nucleotides. Overall, Uracil was the most observed nucleotide, and Adenine was the least observed one with 27.8%, and 22.6%, respectively. 

In the first nucleotide position, 183 miRNAs have an A and 186 miRNAs start with a U. There is a higher frequency of Adenines at the beginning of the sequences when compared to Uracil which is more frequent at the end of miRNA sequences. In particular, there is a high density of Uracils between nucleotide positions 22 and 25. Purine (A and G) and pyrimidine (C and U) nucleotide bases were analyzed for their frequency in the studied miRNA structures (Supplementary Table S2). The highest and lowest purine-scoring miRNAs are listed in Table 3. hsa-mir-765 (85.71% purines), hsa-mir-1468 (81.82%), and hsa-mir-1910 (80.00%) are some of the very high purine content miRNAs. The lowest purine content is present in hsa-mir-1281 (5.88%) and hsa-mir-483 (9.52%).

### 3.2. Motifs in microRNA Sequences Implicated in Myeloid Cancer

In addition to miRNA structure analysis, their common motifs were determined according to their length and frequencies. To find the most abundant motifs in miRNA sequences, we searched for nucleotide motifs containing 3, 4, 5, 6, and 7 nucleotides shared among all miRNA sequences. For this, the smallest motif encoding an amino acid, 3-nucleotide (3n), was searched. Then, the same analysis was done in the form of 4n, 5n, 6n, and 7n.

#### 3.2.1. 3n miRNA Motifs

In the first round of motif search, we analyzed the sequences for 3n motifs, which are the smallest significant motifs. The most observed and the shortest motifs in the miRNA sequences were CUG, UGC, UGG, UGU, CAG, UUG, CCU, CUU, GUG, AGG, UCU, GCU, CGU, CGC, GCG, UGC, ACG, and CGA. These were found in 91.65% of miRNAs. Only 8.35% did not have any of these 3n motifs in their sequences (for example, hsa-mir-122 and hsa-mir-1181) (Table 4).

#### 3.2.2. 4n miRNA Motifs

We divided the 4n motifs identified into two groups, the ones occurring in more than 75 miRNAs (the most detected) and the ones occurring in less than 10 miRNAs (the least detected) (Table 5). 

CUGC, ACUG, and UGCA were found as the most detected motifs in 87 and 85 different miRNAs, respectively. On the other hand, CGAA, CGAG, CGUA, and UCGA were the least detected 4n motifs in 10 different miRNA sequences. Moreover, 112 sequences of 587 microRNAs (19%) do not have any of the top 4n motifs. In addition, 28 of 112 sequences have the least common motifs, and 84 of the miRNAs do not have any of the listed motifs (Supplementary Table S3).

#### 3.2.3. Longer Motifs

The purpose of finding longer motifs such as 5n, 6n, and 7n new motifs was to find potentially conserved or master sites in miRNAs. A total of 271 different 5n motifs were detected (Supplementary Table S3). AGUGC was the most frequent 5n motif found in 36 miRNAs (6%). A total of 38 different 5n motifs were unique (Table 6). The other mostly detected long motifs are made of 6n and 7n sequences. The most frequently observed 6n motifs were AAGUGC and GCUUCC (detected in 22 different miRNAs, 4%), while UUUAGAG was the most detected 7n motif in our dataset (found in 19 miRNAs, 3%). Finally, the longest motifs were detected, 8n (AAGUGCUU), 9n (AAGUGCUUC), 10n (AAAGUGCUUC), and 20n (AAAGUGCUUCCCUUUAGAGU). hsa-mir-106a, hsa-mir-302a, b, c, d, e, hsa-mir-526b have the 8n, 9n, and 10n motifs, but hsa-mir-520a, b, c, d, e, g, and h include all the long motifs in their structures (Supplementary Table S3). 

#### 3.2.4. Consensus miRNA Sequences Having Many Motifs 

Consensus motifs were analyzed in the miRNA sequences, elucidating the consecutive alignment of our motifs in different miRNA sequences to different degrees. In this way, detailed results were obtained about where the identified motifs are located in the miRNA, and how they appear in high-consensus sequences (Table 7).

The results of this analysis show that a miRNA can be associated with one or more mRNA targets, using the common motifs it has in the sequence. Apart from the importance of motifs and consensus sequences in the miRNA binding on their target, the secondary importance of our results may arise in these sequences being a target of RNA binding proteins (RBPs), which recognize specific sequence motifs and are key factors to regulate the miRNA function. Although the transcription factors and epigenetic modifications control the synthesis of miRNAs, their regulation after synthesis is highly controlled with RBPs [32]. Overall studies regarding the RBP binding and regulation of miRNAs are insufficient. Among more than 500 identified human RBPs, only a few have been characterized in terms of functioning in oncogene and tumor suppressor mRNAs [33]. There are many secrets to be revealed behind the miRNA processing by RBPs in healthy and disease states for research to be carried out in the future. The complexity of regulation is further increased with the clues on the cooperative work of miRNAs and RBPs in controlling common mRNA targets [34]. Taking all these into account, a detailed study of the structure of these short RNA molecules, which can perform so many functions, is crucial, and the results presented in our study, can serve as a starting point and raw material for these studies, especially in cancer models.

#### 3.2.5. Target Genes of 7n Motifs

We next analyzed the target genes of the miRNAs that share common motifs, if they give hints on the functional aspects of the motifs we identified in myeloid cancer. For this study, 7n motifs were selected as they are longer and can be more specific in their targets [1]. Using the miRNA-target prediction tool MirTar database, we identified the targets of our miRNAs, which are experimentally validated in different studies. The list of overlapping genes is listed in Table 8, and all the detected targets are given in Supplementary Table S4. 

Our 7n motif GUGCUUC is present in 15 different miRNA sequences and all of them target the same six genes (*EIF2S1*, *SPRED1*, *HIP1*, *YOD1*, *ELK4*, *ABHD15*). We see that this is the case for many motifs in different degrees. This presumes that the motifs which are identified are an important factor for target recognition and small changes in the sequences can impact the specificity of binding/regulation.

#### 3.2.6. Conserved 5n and 6n Motifs

We further wanted to test our motif-finding script in analyzing the conserved miRNA motifs in different species. For this, 5n and 6n motifs were searched in the available miRNA sequences from different species; 645 miRNAs for *Pongo pygmaeus*, 1978 for *Mus musculus*, 437 for *Caenorhabditis elegans*, 690 for *Equus caballus*, 469 for *Drosophila melanogaster*, 600 for *Picea abies*, 695 for *Oreochromis niloticus,* 1138 for *Monodelphis domestica*, 1232 for *Gallus gallus*, 548 for *Ciona intestinalis*, and 2654 for humans (Supplementary Table S5). Among the top 15 identified motifs, we combined the ones that were common to all species and derived their percentages for specific species studied (Table 9).

MiRNAs are key regulators of many cellular processes and may be one of the main players in post-transcriptional regulation. Because they also influence vital biological processes, they tend to be conserved between species. However, there have been contradictory reports on the SNP density of these regions when compared to control [35]. Here, we show that the conservation may happen with certain motifs inside the miRNAs and the higher SNP density may be present in other parts of the miRNA, which add to the list of target genes of miRNA without disturbing the main target interactions.

## 4. Discussion

More than 50% of human genes are predicted to be regulated by miRNAs [36]. They are powerful post-transcriptional modulators of mRNA translation that are proven to regulate many important processes in cancer progression as well [37]. There are more than 70 disease studies that associate with miRNAs [38]. Some of them target the oncogene products and the others the tumor suppressor gene products [39]. Acute myeloid leukemia is a disease characterized by the buildup of immature myeloid cells, mainly resulting from the genetic background. However, the emerging field of miRNA research has already identified certain miRNA profiles behind the disease that correlate with prognosis [40]. Such studies were generally concentrated on the functional effects of specific miRNAs. In our study, we have a general look at the structural aspects shared by miRNAs elevated in AML patients. We addressed different aspects of the structure of the miRNAs up- or downregulated in myeloid cancer patients. 

The first aspect we looked at was the length distribution of miRNAs studied, which had a mode and median of 22 nucleotides, the same as found by a previous study done on overall human miRNAs [1]. In the same study, it was shown that the distribution does not follow a Poisson distribution where the mean and variance would be equal, but rather a Laplace distribution fits better. Next, our analysis of the nucleotide distribution in every position implied the existence of structural patterns in the miRNA sequences. There is a higher occupancy of Adenines at the beginning of the sequences and of Uracil at the end of the sequences. This multinomial distribution of nucleotides in different positions was also noted as significant in the work of Fang et al. for the overall miRNAs studied, except that they did not find a significant difference between the GC and AU content of their samples. In our miRNA dataset of myeloid cancer patients, there was a grouping of miRNAs based on their purine and pyrimidine content, which further supports the pattern presence in their sequences.

MiRNAs can have many mRNA targets due to their ability to exert their function even in imperfect base-pairing. Fang et al. found a positive correlation between the average miRNA length and the number of targets, which may be explained by the higher affinity of longer miRNAs with their targets [1]. In our study, we elucidated a way to look into the motifs that target the same genes. Further research should follow for the functional aspects of this way of the importance of such targeting by many miRNAs using the same motifs on the same genes.

Some miRNAs were shown to have conserved functions beginning from mosses and ferns [41]. In a study by Vazquez et al., longer miRNAs were shown to have a more recent history in Arabidopsis. They also found a correlation between the bases at certain miRNA sites to be conserved [42]. In another study by Lewis et al., they noted that the nucleotide position upstream and downstream of seed regions of miRNAs were highly conserved [43]. In our research, we have shown that the conservation of certain motifs is present between different species to different degrees.

Overall, our research may serve as a strategy to study the common structural aspects of miRNAs in different human diseases. Furthermore, it can be extended to study the functional outcomes of the presence of motifs and more cancer types to produce an inclusive comparative study.

## 5. Conclusions

In conclusion, this research reveals motif sequences of miRNAs implicated in myeloid cancer, which were also shown to be clustered in consensus sequences. Moreover, it was shown that many of these motifs tend to be conserved across species.

## Figures and Tables

**Table 1:** Nucleotide sequences of miRNAs implicated in myeloid cancer ^1^.

	5′	Direction																									3′	
	1	2	3	4	5	6	7	8	9	10	11	12	13	14	15	16	17	18	19	20	21	22	23	24	25	26	27	Length
hsa-mir-1248	A	C	C	U	U	C	U	U	G	U	A	U	A	A	G	C	A	C	U	G	U	G	C	U	A	A	A	27
hsa-mir-1183	C	A	C	U	G	U	A	G	G	U	G	A	U	G	G	U	G	A	G	A	G	U	G	G	G	C	A	27
hsa-mir-1272	G	A	U	G	A	U	G	A	U	G	G	C	A	G	C	A	A	A	U	U	C	U	G	A	A	A		26
hsa-mir-1244	A	A	G	U	A	G	U	U	G	G	U	U	U	G	U	A	U	G	A	G	A	U	G	G	U	U		26
hsa-mir-921	C	U	A	G	U	G	A	G	G	G	A	C	A	G	A	A	C	C	A	G	G	A	U	U	C			25
hsa-mir-638	A	G	G	G	A	U	C	G	C	G	G	G	C	G	G	G	U	G	G	C	G	G	C	C	U			25
…																												
hsa-mir-1279	U	C	A	U	A	U	U	G	C	U	U	C	U	U	U	C	U											17

^1^ Position 1–27 corresponds to direction 5′-3′. The complete data can be found in Supplementary Table S1.

**Table 2:** Comparison of short and long miRNA publication numbers. Retrieved in February 2022 from reference counts of each miRNA page in mirbase.org.

Short miRNAs	Nucleotide Length	Publications
hsa-mir-1275	17	33
hsa-mir-302e	17	160
hsa-mir-1207	18	33
**Long miRNAs**	**Nucleotide Length**	**Publications**
hsa-mir-1248	27	16
hsa-mir-1183	27	5
hsa-mir-1272	26	5
hsa-mir-1244	26	7

**Table 3:** Purine rich and poor miRNAs.

miRNA	Highest Purine Content	A	G	C	U	%A + G
hsa-mir-765	UGGAGGAGAAGGAAGGUGAUG	7	11	0	3	85.71
hsa-mir-1468	AGCAAAAUAAGCAAAUGGAAAA	14	4	2	2	81.82
hsa-mir-1910	GAGGCAGAAGCAGGAUGACA	8	8	3	1	80.00
hsa-mir-202	AGAGGUAUAGGGCAUGGGAA	7	9	1	3	80.00
hsa-mir-1255a	AGGAUGAGCAAAGAAAGUAGAUU	11	7	1	4	78.26
hsa-mir-320a	AAAAGCUGGGUUGAGAGGGCGA	7	10	2	3	77.27
hsa-mir-936	ACAGUAGAGGGAGGAAUCGCAG	8	9	3	2	77.27
hsa-mir-149	AGGGAGGGACGGGGGCUGUGC	3	13	3	2	76.19
	Lowest purine content					
hsa-mir-1281	UCGCCUCCUCCUCUCCC	0	1	11	5	5.88
hsa-mir-483	UCACUCCUCUCCUCCCGUCUU	1	1	11	8	9.52
hsa-mir-877	UCCUCUUCUCCCUCCUCCCAG	1	1	12	7	9.52
hsa-mir-1236	CCUCUUCCCCUUGUCUCUCCAG	1	2	11	8	13.64
hsa-mir-1249	ACGCCCUUCCCCCCCUUCUUCA	2	1	13	6	13.64
hsa-mir-1224	CCCCACCUCCUCUCUCCUCAG	2	1	13	5	14.29
hsa-mir-1238	CUUCCUCGUCUGUCUGCCCC	0	3	10	7	15.00

**Table 4:** The list of identified 3n motifs in studied myeloid cancer miRNA dataset.

3n Motif	Frequency	Percentage
CUG	249	42.42%
UGC	234	39.86%
UGG	233	39.69%
UGU	231	39.35%
CAG	213	36.29%
UUG	213	36.29%
CCU	205	34.92%
CUU	205	34.92%
GUG	202	34.41%
AGG	196	33.39%
UCU	195	33.22%
GCU	191	32.54%
CGU	74	12.61%
CGC	72	12.27%
GCG	66	11.24%
UCG	62	10.56%
ACG	56	9.54%
CGA	42	7.16%

**Table 5:** The number of most and least detected 4n motifs.

Most Detected 4n Motifs			Least Detected 4n Motifs		
Present in >70 miRNAs			Present in <10 miRNAs		
Motif	Frequency	Percentage	Motif	Frequency	Percentage
CUGC	87	14.82%	CGAA	10	1.70%
ACUG	85	14.48%	CGAG	10	1.70%
UGCA	85	14.48%	CGUA	10	1.70%
CUUU	83	14.14%	UCGA	10	1.70%
AGUG	82	13.97%	ACGA	9	1.53%
CUGG	80	13.63%	ACGC	9	1.53%
CUGU	80	13.63%	UACG	8	1.36%
UUUG	79	13.46%	CGAU	7	1.19%
CAGU	78	13.29%	UUCG	6	1.02%
UUCU	78	13.29%			
UGCU	77	13.12%			
UGUG	77	13.12%			
GUGC	76	12.95%			
UGGG	76	12.95%			
UCUG	75	12.78%			

**Table 6:** The highly observed long motifs.

7n Motif	Frequency	6n Motif	Frequency	5n Motif	Frequency	4n Motif	Frequency
UUUAGAG	19	AAGUGC	22	AGUGC	36	CUGC	87
AAGUGCU	18	GCUUCC	22	CUUCC	34	ACUG	85
AGUGCUU	16	UUUAGA	21	GCUUC	33	UGCA	85
GUGCUUC	15	UUAGAG	20	AAGUG	32	CUUU	83
UGCUUCC	15	UGCUUC	19	CCUUU	32	AGUG	82
		AGUGCU	18	CUGCC	31	CUGG	80

**Table 7:** Motifs and consensus sequences in miRNAs.

miRNA	5′-3′	3n	4n	5n	6n	7n
519b 520c 526b	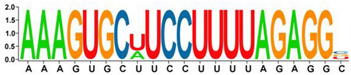	GUG	GAGG	AGUGC	AAAGUG	UUUAGAG
		UGC	CUUU	CUUUU	AAGUGC	AAAGUGC
		CCU	GUGC	AAGUG	UUUAGA	UCCUUUU
		CUU	UAGA	AGAGC	UUAGAG	UUAGAGG

**Table 8:** MiRNA with 7n motifs and their gene targets.

7n Motif	Frequency	Targeted Gene	Frequency	Percentage
GUGCUUC	15	*EIF2S1*	15	100
GUGCUUC	15	*SPRED1*	15	100
GUGCUUC	15	*HIP1*	15	100
GUGCUUC	15	*YOD1*	15	100
GUGCUUC	15	*ELK4*	15	100
GUGCUUC	15	*ABHD15*	15	100
UGCUUCC	15	*ACOX1*	14	93.33
UGCUUCC	15	*EIF2S1*	13	86.67
UGCUUCC	15	*HOOK3*	13	86.67
UGCUUCC	15	*SPRED1*	13	86.67
UGCUUCC	15	*HIP1*	13	86.67
UGCUUCC	15	*YOD1*	13	86.67
AGUGCUU	16	*PNRC1*	15	93.75
AGUGCUU	16	*DNAJC10*	15	93.75
AGUGCUU	16	*HMGB1*	15	93.75
AGUGCUU	16	*MED18*	15	93.75
AGUGCUU	16	*MASTL*	15	93.75
AGUGCUU	16	*DSTYK*	15	93.75
AAGUGCU	18	*HMGB1*	17	94.44
AAGUGCU	18	*PNRC1*	16	88.89
AAGUGCU	18	*DNAJC10*	16	88.89
AAGUGCU	18	*MED18*	16	88.89
AAGUGCU	18	*MASTL*	16	88.89
AAGUGCU	18	*DSTYK*	16	88.89
UUUAGAG	19	*YOD1*	8	42.11
UUUAGAG	20	*ABHD15*	8	40.00
UUUAGAG	21	*DNAJC28*	8	38.10
UUUAGAG	22	*SAMD8*	8	36.36
UUUAGAG	23	*PRRG4*	8	34.78
UUUAGAG	24	*GPR157*	8	33.33

**Table 9:** 5n and 6n motifs conserved between humans and other species.

	5n Motifs						6n Motifs		
	CAGUG	CUGGG	UGCAG	UUUUC	UUGCA	GAGAG	AGUGCU	AGUGCA	CUGCAG
*Pongo pygmaeus*	5.4%	4.8%	4.8%				3.1%	2.9%	
*Mus musculus*	5.2%	5.7%	5.0%						2.1%
*Equus caballus*	6.2%	6.4%	5.8%				2.0%	2.3%	2.3%
*Oreochromis niloticus*	6.6%		6.9%					2.9%	3.2%
*Monodelphis domestica*	4.5%						2.0%	1.8%	
*Gallus gallus*	5.8%		7.2%				2.4%		3.0%
Human		6.0%	5.2%						2.2%
*Caenorhabditis elegans*				7.3%					
*Drosophila melanogaster*					8.3%				
*Picea abies*						10.7%			

## Data Availability

Data is available online (https://doi.org/10.6084/m9.figshare.20152466) or upon request from the corresponding author.

## Supplementary Materials

The following supporting information can be downloaded at: https://www.mdpi.com/article/10.3390/genes13071152/s1. Table S1: List of all identified miRNAs implicated in myeloid cancer; Table S2: Purine and py-rimidine analysis of all miRNAs; Table S3: Motif analysis results of miRNAs studied; Table S4: Target gene analysis for all miRNAs; Table S5: Conserved motif analysis among species.
